# Supplementary material for: Metabolomics Analysis Reveals Molecular Signatures of Metabolic Complexity in Children with Hypercholesterolemia
Source: Nutrients. 2023 Mar 31;15(7):1726. doi: 10.3390/nu15071726 (PMC10096550; doi:10.3390/nu15071726)
Supplement: Supplementary file 1 [file nutrients-15-01726-s001.zip › Supplementary file.pdf]

Supplementary Figures and Tables

TABLE S1 PLS-DA parameters and permutation test for distinguishing between children with different status of cholesterol level.

	PLS-DA parameters				
Cholesterol level status	Components*	Q^2	R^2	Q^2/R^2	$P_{permutation}$†
High vs. Acceptable	1	-0.05	0.19	-0.25	0.161
Borderline vs. Acceptable	3	-0.61	0.28	-1.54	0.170
High vs. Borderline	1	0.00	0.13	0.01	0.378

PLS-DA, partial least squares-discriminant analysis; Q^2, predictive capability; R^2, correlation coefficients. *The number of components based on Q^2 indicates the best classifier of PLS-DA using a 10-fold cross-validation method. †1000 random permutations were performed.

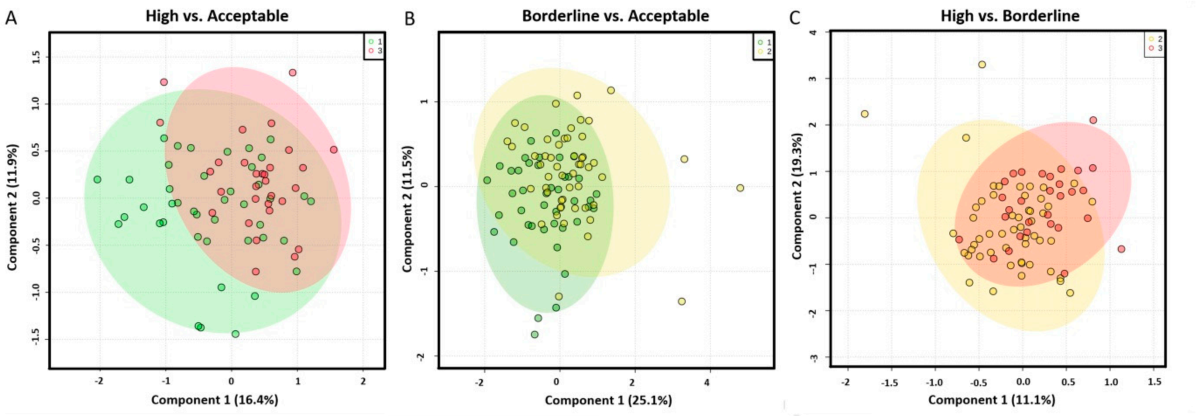

Figure S1 PLS-DA score plots from the analysis of plasma ^{1}H-NMR spectra between high and acceptable levels of cholesterol (A), and between borderline and acceptable levels of cholesterol (B), and between high and borderline levels of cholesterol (C). 1, acceptable cholesterol level; 2, borderline cholesterol level; 3, high cholesterol level; x axis, component 1 (% of total variance); y axis, component 2 (% of total variance).
